# Supplementary material for: Diverse and abundant phages exploit conjugative plasmids
Source: Nat Commun. 2024 Apr 12;15:3197. doi: 10.1038/s41467-024-47416-z (PMC11015023; doi:10.1038/s41467-024-47416-z)
Supplement: Supplementary file 3 — Description of Additional Supplementary Files [file 41467_2024_47416_MOESM3_ESM.pdf]

Description of Additional Supplementary Files:

Supplementary Dataset 1: Spreadsheet listing all bacterial strains, plasmids, phages, and primers and environmental samples used in this study.

Supplementary Dataset 2: Spreadsheet listing all phage genomes used for comparative genomics analyses (tectiviruses, fiersviruses, inoviruses, FtMidnight-relatives), and SRA datasets for metagenomic analyses.

Supplementary Dataset 3: Spreadsheet listing CRISPR-Cas and RM hits in bacterial hosts.
