# Supplementary material for: Diverse and abundant phages exploit conjugative plasmids
Source: Nat Commun. 2024 Apr 12;15:3197. doi: 10.1038/s41467-024-47416-z (PMC11015023; doi:10.1038/s41467-024-47416-z)
Supplement: Supplementary file 7 — Reporting Summary [file 41467_2024_47416_MOESM7_ESM.pdf]

nature portfolio

Corresponding author(s): Michael Baym
Siân Owen

Last updated by author(s): Apr 19, 2023

Reporting Summary

Nature Portfolio wishes to improve the reproducibility of the work that we publish. This form provides structure for consistency and transparency in reporting. For further information on Nature Portfolio policies, see our Editorial Policies and the Editorial Policy Checklist.

Statistics

For all statistical analyses, confirm that the following items are present in the figure legend, table legend, main text, or Methods section.

n/a	Confirmed	
☐	☒	The exact sample size (*n*) for each experimental group/condition, given as a discrete number and unit of measurement
☐	☒	A statement on whether measurements were taken from distinct samples or whether the same sample was measured repeatedly
☒	☐	The statistical test(s) used AND whether they are one- or two-sided<br>*Only common tests should be described solely by name; describe more complex techniques in the Methods section.*
☒	☐	A description of all covariates tested
☒	☐	A description of any assumptions or corrections, such as tests of normality and adjustment for multiple comparisons
☐	☒	A full description of the statistical parameters including central tendency (e.g. means) or other basic estimates (e.g. regression coefficient) AND variation (e.g. standard deviation) or associated estimates of uncertainty (e.g. confidence intervals)
☒	☐	For null hypothesis testing, the test statistic (e.g. *F*, *t*, *r*) with confidence intervals, effect sizes, degrees of freedom and *P* value noted<br>*Give P values as exact values whenever suitable.*
☒	☐	For Bayesian analysis, information on the choice of priors and Markov chain Monte Carlo settings
☒	☐	For hierarchical and complex designs, identification of the appropriate level for tests and full reporting of outcomes
☒	☐	Estimates of effect sizes (e.g. Cohen's *d*, Pearson's *r*), indicating how they were calculated

Our web collection on statistics for biologists contains articles on many of the points above.

Software and code

Policy information about availability of computer code

Data collection	Custom code used has been made publicly available on github as indicated in the manuscript https://github.com/baymlab/2023_QuinonesOlvera-Owen<br>Software used for data collection:<br>scikit-image v0.17.2
Data analysis	Custom code used has been made publicly available on github as indicated in the manuscript https://github.com/baymlab/2023_QuinonesOlvera-Owen<br>Software used for data analysis:<br>prokka v1.14.6<br>clinker v1.0<br>HHpred via MPI Bioinformatics Toolkit<br>tblastn<br>CRISPRCasTyper v1.8.0<br>DefenseFinder v1.0.9<br>HMMER v3.3.1<br>Kraken2 v2..1.2<br>samtools v1.6<br>IGV v2.11.4<br>iqtree v2.2.0.3<br>iqtree v2.2.0.3

iTOL v6.7
phyml v3.2.0
FigTree v1.4.4
Trimmomatic v0.39
Rasusa v0.5.0
Unicycler v0.4.8
rnaviralSPADES v.3.15.5
RATT v.1.0.3
minimap2 v2.24
bcftools v.1.9
samtools v1.6
vcftools 0.1.16
seaborn v0.12.2
matplotlib v1.0
Protter v1.0
CCTOP v1.1.0
ColabFold v1.5.3
Pymol v2.5.6
clustalo v1.2.4
UGENE v38.1

For manuscripts utilizing custom algorithms or software that are central to the research but not yet described in published literature, software must be made available to editors and reviewers. We strongly encourage code deposition in a community repository (e.g. GitHub). See the Nature Portfolio guidelines for submitting code & software for further information.

Data

Policy information about availability of data

All manuscripts must include a data availability statement. This statement should provide the following information, where applicable:

- Accession codes, unique identifiers, or web links for publicly available datasets
- A description of any restrictions on data availability
- For clinical datasets or third party data, please ensure that the statement adheres to our policy

Raw sequencing reads have been deposited in the NCBI BioProject database under accession number PRJNA954020.
Genome accession numbers used: in this work:
KJ802832.1
PP430153.1
NC_001421.2
V00604.2
V00642.1
AY099114.1
PP412815.1
PP412816.1
PP412817.1
PP412818.1
PP412819.1
PP412820.1
PP412821.1
PP412822.1
PP412823.1
PP412824.1
PP412825.1
PP412826.1
PP412827.1
PP412828.1
PP412829.1
PP412830.1
PP412831.1
PP412832.1
PP412833.1
PP412834.1
PP412835.1
PP412836.1
PP412837.1
PP412838.1
PP412839.1
PP412840.1
PP412841.1
PP412842.1

PP412843.1
PP412844.1
PP412845.1
PP412846.1
PP412847.1
PP412848.1
PP412849.1
PP412850.1
PP412851.1
PP412852.1
PP412853.1
PP412854.1
PP412855.1
PP412856.1
PP412857.1
PP412858.1
PP412859.1
PP412860.1
PP412861.1
PP412862.1
PP412863.1
PP412864.1
PP412865.1
PP430139.1
PP430142.1
PP430140.1
PP430143.1
PP430144.1
PP430141.1
PP430145.1
PP430147.1
PP430148.1
PP430149.1
PP430150.1
PP430151.1
PP430152.1
PP430146.1

SRA run accessions:
SRR24145760
SRR24145729
SRR24145730
SRR24145754
SRR24145732
SRR24145735
SRR24145731
SRR24145768
SRR24145770
SRR24145753
SRR24145761
SRR24145708
SRR24145752
SRR24145755
SRR24145734
SRR24145747
SRR24145771
SRR24145746
SRR24145744
SRR24145767
SRR24145727
SRR24145757
SRR24145709
SRR24145762
SRR24145769
SRR24145749
SRR24145756
SRR24145726
SRR24145764

SRR24145707
SRR24145738
SRR24145733
SRR24145750
SRR24145745
SRR24145742
SRR24145736
SRR24145766
SRR24145741
SRR24145739
SRR24145737
SRR24145716
SRR24145758
SRR24145763
SRR24145743
SRR24145725
SRR24145728
SRR24145740
SRR24145765
SRR24145759
SRR24145751
SRR24145748
SRR28247442
SRR28247440
SRR28247441
SRR28247439
SRR28247444
SRR28247438
SRR28247443
SRR24145712
SRR24145713
SRR24145714
SRR24145715
SRR24145717
SRR24145711
SRR28248334

Research involving human participants, their data, or biological material

Policy information about studies with human participants or human data. See also policy information about sex, gender (identity/presentation), and sexual orientation and race, ethnicity and racism.

Reporting on sex and gender	N/A
Reporting on race, ethnicity, or other socially relevant groupings	N/A
Population characteristics	N/A
Recruitment	N/A
Ethics oversight	N/A

Note that full information on the approval of the study protocol must also be provided in the manuscript.

Field-specific reporting

Please select the one below that is the best fit for your research. If you are not sure, read the appropriate sections before making your selection.

[x] Life sciences [ ] Behavioural & social sciences [ ] Ecological, evolutionary & environmental sciences

For a reference copy of the document with all sections, see nature.com/documents/nr-reporting-summary-flat.pdf

Life sciences study design

All studies must disclose on these points even when the disclosure is negative.

Sample size	Sample sizes were limited by availability, e.g. only two independent sources of wastewater were routinely available to us. Where we had control over sample sizes, data was always collected from 3 or more independent samples to maximize data accuracy
Data exclusions	No data were excluded from the analyses
Replication	Data were collected in replicate to maximize reproducibility. Number of replicates was often limited by technical constraint but where averages were used replicate number was always >3
Randomization	Randomization was not applicable in this study as the data we collected was not susceptible to selection bias
Blinding	Blinding was not applicable in this study as the data we collected was not susceptible to observer bias

Reporting for specific materials, systems and methods

We require information from authors about some types of materials, experimental systems and methods used in many studies. Here, indicate whether each material, system or method listed is relevant to your study. If you are not sure if a list item applies to your research, read the appropriate section before selecting a response.

Materials & experimental systems

n/a	Involved in the study	
☒	☐	Antibodies
☒	☐	Eukaryotic cell lines
☒	☐	Palaeontology and archaeology
☒	☐	Animals and other organisms
☒	☐	Clinical data
☒	☐	Dual use research of concern
☒	☐	Plants

Methods

n/a	Involved in the study	
☒	☐	ChIP-seq
☒	☐	Flow cytometry
☒	☐	MRI-based neuroimaging
